# Analysis of Patient Narratives in Disease Blogs on the Internet: An Exploratory Study of Social Pharmacovigilance

**DOI:** 10.2196/publichealth.6872

**Published:** 2017-02-24

**Authors:** Shinichi Matsuda, Kotonari Aoki, Shiho Tomizawa, Masayoshi Sone, Riwa Tanaka, Hiroshi Kuriki, Yoichiro Takahashi

**Affiliations:** ^1^ Chugai Pharmaceutical Co Ltd Drug Safety Data Management Department Tokyo Japan; ^2^ Chugai Pharmaceutical Co Ltd Pharmacovigilance Department Tokyo Japan; ^3^ Chugai Pharmaceutical Co Ltd Medical Information Department Tokyo Japan; ^4^ Chugai Pharmaceutical Co Ltd Clinical Science & Strategy Department Tokyo Japan

**Keywords:** Internet, social media, adverse drug reaction, pharmacovigilance, text mining

## Abstract

**Background:**

Although several reports have suggested that patient-generated data from Internet sources could be used to improve drug safety and pharmacovigilance, few studies have identified such data sources in Japan. We introduce a unique Japanese data source: *tōbyōki*, which translates literally as “an account of a struggle with disease.”

**Objective:**

The objective of this study was to evaluate the basic characteristics of the TOBYO database, a collection of *tōbyōki* blogs on the Internet, and discuss potential applications for pharmacovigilance.

**Methods:**

We analyzed the overall gender and age distribution of the patient-generated TOBYO database and compared this with other external databases generated by health care professionals. For detailed analysis, we prepared separate datasets for blogs written by patients with depression and blogs written by patients with rheumatoid arthritis (RA), because these conditions were expected to entail subjective patient symptoms such as discomfort, insomnia, and pain. Frequently appearing medical terms were counted, and their variations were compared with those in an external adverse drug reaction (ADR) reporting database. Frequently appearing words regarding patients with depression and patients with RA were visualized using word clouds and word cooccurrence networks.

**Results:**

As of June 4, 2016, the TOBYO database comprised 54,010 blogs representing 1405 disorders. Overall, more entries were written by female bloggers (68.8%) than by male bloggers (30.8%). The most frequently observed disorders were breast cancer (4983 blogs), depression (3556), infertility (2430), RA (1118), and panic disorder (1090). Comparison of medical terms observed in *tōbyōki* blogs with those in an external ADR reporting database showed that subjective and symptomatic events and general terms tended to be frequently observed in *tōbyōki* blogs (eg, anxiety, headache, and pain), whereas events using more technical medical terms (eg, syndrome and abnormal laboratory test result) tended to be observed frequently in the ADR database. We also confirmed the feasibility of using visualization techniques to obtain insights from unstructured text-based *tōbyōki* blog data. Word clouds described the characteristics of each disorder, such as “sleeping” and “anxiety” in depression and “pain” and “painful” in RA.

**Conclusions:**

Pharmacovigilance should maintain a strong focus on patients’ actual experiences, concerns, and outcomes, and this approach can be expected to uncover hidden adverse event signals earlier and to help us understand adverse events in a patient-centered way. Patient-generated *tōbyōki* blogs in the TOBYO database showed unique characteristics that were different from the data in existing sources generated by health care professionals. Analysis of *tōbyōki* blogs would add value to the assessment of disorders with a high prevalence in women, psychiatric disorders in which subjective symptoms have important clinical meaning, refractory disorders, and other chronic disorders.

## Introduction

### Current Pharmacovigilance

The World Health Organization defines pharmacovigilance (PV) as the science and activities related to the detection, assessment, understanding, and prevention of adverse effects or any other drug-related problems [1]. In this era of what Edwards calls “information explosion,” we must rethink PV [2] to effectively incorporate a variety of data sources while ensuring the timely decision-making that is crucial to avoiding unnecessary harm caused by adverse events (AEs) in real-world health care practice.

Current PV activities depend heavily on voluntary, spontaneous AE reports obtained from health care professionals (HCPs). It is generally accepted that one advantage of spontaneous reporting is its speed at detecting AE signals as early as possible. However, it is also acknowledged that spontaneous reports by HCPs alone may not be enough to capture all AE signals in a timely fashion. Because some symptomatic AEs can be expected to be reported only by patients who have firsthand experience of drug treatment [3], incorporating patient-generated data into PV is one of the most important challenges [4]. Several studies have suggested that self-reporting by patients is useful for catching AE signals earlier, and many countries have implemented patient AE reporting schemes [5-8]. The Japanese regulatory authority started preliminary implementation of a self-reporting system for patients in March 2012 [9,10]; however, the system is still under development and will require more time to be used effectively in a routine PV system [11].

### Prior Research on Applying Internet Resources in Pharmacovigilance

Analyzing information on the Internet would add significant knowledge about public health, as shown in Eysenbach’s study outlining the framework of infodemiology and infoveillance [12]. In PV, there has been recent growing interest in utilizing patient-generated Internet resources such as social media [13-17]. A survey conducted in 2001 and 2002 in the United States showed that the Internet is an important resource for the public; approximately 40% of respondents there obtained information on health-related topics through Internet sources [18]. In response to the increasing use of social media to share health care information, the US Food and Drug Administration announced in 2015 that they had started a collaboration with PatientsLikeMe [19], a patient networking website, to apply patient-generated data to risk management activities [20]. In Europe, the Medicines and Healthcare products Regulatory Agency in the United Kingdom started working with the WEB-RADR project in 2014 to develop a mobile phone app that helps HCPs and patients report AEs to national health care authorities [21]. The European Medicines Agency has also released guidelines on good pharmacovigilance practices, of which Module VI requires companies having the European Union marketing authorization to monitor the Internet or digital media under their management or responsibility for potential reports of suspected adverse reactions [22]. These ongoing efforts are expected to lead to important developments in PV. Like Americans and Europeans, approximately 39% of Japanese obtain health information via the Internet [23]. However, to our knowledge, no studies have explicitly identified such Japanese data sources for use in PV.

### Patient-Generated Data and Study Objectives

Our motivation was to take the first step toward enhancing PV by considering the application of patient-generated data sources in Japan. In this study, we focused on the potential use of health-related disease blogs called *tōbyōki*. The term *tōbyōki* translates literally to “an account of a struggle with disease,” and this form of writing predates the Internet. Although it is difficult to pinpoint when patients started writing *tōbyōki*, a sociological study has reported that the number of *tōbyōki* has been increasing in Japan since the 1970s [24]. In these diary-like accounts, patients record observations about their lives and diseases in handwritten journals. Recently, some patients have started sharing their *tōbyōki* as blogs on the Internet.

It has already been suggested that analyzing *tōbyōki* blogs is useful for understanding patients’ feelings when they receive a cancer diagnosis [25], although there was no discussion on their potential use in PV. In this study, we introduce a growing database called TOBYO, which is a collection of a broad range of *tōbyōki* blogs on the Internet [26]. The objective of this exploratory study was to address the following questions: (1) what kinds of data elements exist in the TOBYO database? (2) what are the differences in population distribution between the TOBYO database and other external databases generated by HCPs? (3) what kinds of analytic approaches are useful to obtain insights from the TOBYO database? and (4) can the TOBYO database be useful for PV?

To achieve our objective, we conducted 2 analyses (Analysis A and Analysis B). In Analysis A, we used the whole TOBYO database to describe data elements and understand the overall characteristics of this database. In Analysis B, we used a data subset of selected disorders from the TOBYO database to explore the usefulness of the database in greater detail. Here, we focused on depressive disorders and rheumatoid arthritis (RA) because these conditions were expected to entail subjective patient symptoms such as discomfort, insomnia, and pain. Finally, we included a discussion of the potential of the TOBYO database and practical challenges from the PV perspective.

## Methods

### Data Source

In this study, we considered health-related *tōbyōki* blogs as a resource for patient-generated data. Some examples of excerpts from *tōbyōki* blogs are shown in Table 1. As shown in these examples, patients shared information about AEs, drug name, dosage, and AE-related distress.

**Table 1:** Example of excerpts from *tōbyōki* blog.

Name of disorder	Excerpt from each *tōbyōki* blog^a^
Breast cancer	I showed my leg with a ruptured blister to my doctor. He said it is a side effect of docetaxel and it needs a long time to be cured. Also, I was advised to avoid secondary infection from this site.	
Depression	After changing clinics, I reduced the number of drugs I’m taking. At first I couldn’t help being nervous, but I’m feeling well now; morning milnacipran 15 mg, alprazolam 0.4 mg, mid-afternoon alprazolam 0.4 mg (as needed), evening milnacipran 15 mg, alprazolam 0.4 mg. That’s all.
Rheumatoid arthritis	Thanks to my second dose of etanercept, the pain in my joints is completely gone! Because of this dramatic improvement, my doctor reduced my prescription of methotrexate to 2 mg/day, and also promised to reduce my steroids, too. I am really happy because I can escape from the side effects of steroids, which are the hardest thing for me...

^a^All entries are translated from Japanese. To protect patient privacy, minor changes were made to texts while maintaining the meaning of original contexts.

**Figure 1:**
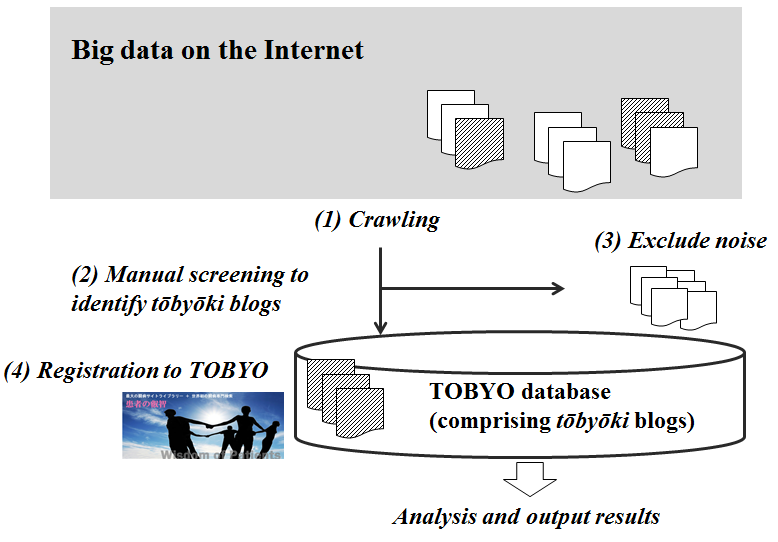
Overall flow of data in the TOBYO database.(1) This study focuses on tōbyōki blogs that are publicly available on the Internet. Generally, there is a substantial volume of noise (white) unrelated to tōbyōki blogs (shaded). (2) Based on selection criteria described in Methods, filtering of tōbyōki blogs is performed manually, (3) and noise such as blogs written by companies is excluded. (4) Appropriate tōbyōki blogs are registered in the TOBYO database and stored for additional analysis.

The TOBYO database consisted of a Web-based collection of *tōbyōki* blogs written in Japanese [26] and maintained by Initiative Inc (Tokyo, Japan). The overall flow of data in the TOBYO database is shown in Figure 1. Blogs written in Japanese were identified and extracted daily from the Internet using a proprietary crawling method. Before being registered in the TOBYO database, each *tōbyōki* blog was manually checked to judge whether it was a *tōbyōki* blog or noise, which was excluded. Each blog registered to the TOBYO database met all of the following selection criteria: (1) Language　criteria: blogs written in plain Japanese language without extensive use of emoticons, symbols, or colloquial expressions were included; (2) Blogger criteria: blogs written by patients or their families were included. Blogs not written by patients or their families, such as those by manufacturers or HCPs who were providing medical care, were excluded (because such blogs generally described the HCP’s records and did not contain a patient perspective); and (3) Content criteria: blogs containing at least ten pages of *tōbyōki* entries on patients’ actual experiences were included. Blogs comprising excerpts from news media, books, health-related websites, or treatment guidelines were excluded. Blogs intended for marketing or promotion of commercial services or religious or political beliefs were also excluded.

At the time of registration in the TOBYO database, information on gender, age at onset, and the primary disorder of each patient was determined by checking the profile or introduction page of each *tōbyōki* blog and stored as structured data for each patient. Text-based data in *tōbyōki* blogs were stored as unstructured data for each patient.

### Analysis A: Using the Whole TOBYO Database

#### Demographic Characteristics of the TOBYO Database

To understand the demographic characteristics of the TOBYO database, structured data elements such as gender, age at onset, and frequently mentioned primary disorders were summarized in contingency tables. We also evaluated demographic characteristics by comparing population pyramids for the TOBYO database and 2 external databases generated by HCPs. The first HCP-generated database was the Japanese Adverse Drug Event Report (JADER) database maintained by the Pharmaceuticals and Medical Devices Agency. It comprised individual case safety reports (ICSRs) about the occurrences of serious adverse drug reactions (ADRs) for drugs approved in Japan. Similar to a previous report [27], we obtained the JADER dataset updated in September 2016 and extracted all ICSRs to create a population pyramid for the JADER database. The other HCP-generated database was the Japanese health insurance claims database maintained by Japan Medical Data Center, Ltd (Tokyo, Japan). It comprised medical claims information submitted from medical institutions to health insurance organizations for both corporate employees and their dependents [28]. Using this database, we created a population pyramid by determining the number of patients who had at least one record of drug prescription or disease from January 2011 to December 2015. As an additional comparison, we used national statistical surveillance data on all citizens living in Japan and publicly available through the Japanese government’s website [29].

#### Distribution of Disorders in the TOBYO Database

To understand the distribution of primary disorders in the TOBYO database, frequently mentioned disorders were summarized. The name of each disorder was independently reviewed by 2 reviewers (ST and MS) and coded using Medical Dictionary for Regulatory Activities (MedDRA) version 19.1. MedDRA is a widely used, standardized medical terminology developed by the International Council for Harmonisation of Technical Requirements for Pharmaceuticals for Human Use [30]. Both reviewers had at least two years of experience in processing and evaluating ICSRs.

#### Additional Characteristics of the TOBYO Database

We analyzed additional characteristics of *tōbyōki* blogs that might be useful to understand the data. Behavioral characteristics about writing *tōbyōki* blogs, such as the time and day of week for blog postings, were determined for all postings accompanied by relevant identifiable information. Continuity of *tōbyōki* blogs was calculated by counting the number of days from the first entry to the latest update for each patient.

### Analysis B: Using Subset of Selected Disorders in the TOBYO Database

#### Mining Events Appearing in Tōbyōki Blogs

**Figure 2:**
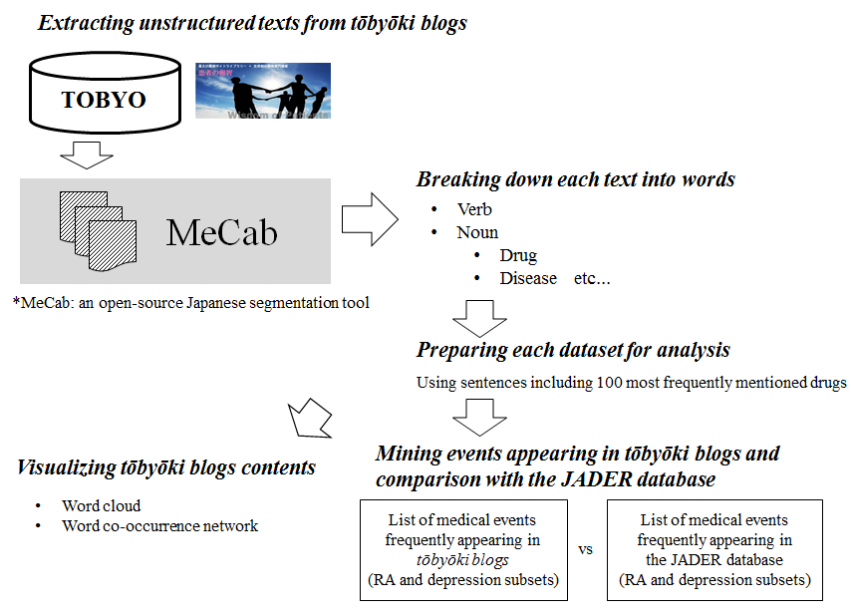
Work flow for morphological analysis and preparation of datasets.

As depicted in Figure 2, we applied natural language processing techniques to unstructured text-based data to prepare each dataset, which were then analyzed to answer specific questions (eg, what identifying words are frequently used by a particular population?). In this study, we extracted 2 different sets of *tōbyōki* blogs from the TOBYO database, 1 for patients with depression and 1 for patients with RA, and we prepared separate datasets containing all unstructured text written by patients with each disorder. We then analyzed the drugs and medical events mentioned in each dataset.

To process the unstructured text, we first performed a morphological analysis using MeCab, an open-source Japanese segmentation tool [31], to break down each text into words. This preprocessing approach is commonly used to delimit words in texts that do not delimit words with spaces, which is a characteristic of the Japanese language [32]. Because *tōbyōki* blogs contained many entries unrelated to disease, such as those related to everyday life, making the data noisy, we also identified the 100 most frequently mentioned drugs in each dataset (depression and RA). Then, for each dataset, we extracted every sentence containing at least one of the 100 most frequently mentioned drugs identified earlier, and these extracted sentences were used for subsequent analysis. This approach enabled us to focus on drug-related contexts rather than on everyday diary-like content. As mentioned earlier, 2 reviewers (ST and MS) independently reviewed summary tables containing the 300 most frequently mentioned words in each dataset (depression and RA) to identify medical events (eg, name of symptom, diagnosis, and disorder), which were coded using MedDRA. Because original descriptions written by patients tended to have some degree of ambiguity (eg, words such as suffering, feeling down, feeling unwell), discrepancies in coding sometimes occurred between the results of the 2 reviewers. The reviewers discussed any such discrepancies and determined a single appropriate Preferred Term in accordance with the standard guidance for MedDRA coding procedures (MedDRA Term Selection: Points to Consider [33]). Any discrepancies in coding results were resolved by discussion.

In addition to identifying medical events frequently observed in *tōbyōki* blogs, we examined differences in the types and frequencies of events between *tōbyōki* blogs and existing HCP-generated data sources. For this purpose, we compared medical terms frequently observed in *tōbyōki* blogs (as identified earlier) and those frequently observed in the JADER database. Using the JADER database, we first produced separate tables of the 30 most frequent ADRs reported for 4 biological drugs approved for RA (adalimumab, etanercept, infliximab, and tocilizumab were selected because they were the first 4 biologics approved in Japan around 2000 and were thus expected to contain enough data for comparison) and that of the 30 most frequent ADRs reported for 4 selective serotonin reuptake inhibitors approved for depression (escitalopram, fluvoxamine, paroxetine, and sertraline were selected because these were widely prescribed and also used in the previous study [14]). Then by comparing these lists of events from *tōbyōki* blogs and the JADER database, we identified the words appearing in both databases and those appearing in either database. This focused comparison based on frequently appearing events enabled us to highlight the major characteristics of these databases. This process of review and comparison was carried out independently by the 2 aforementioned reviewers (ST and MS).

#### Visualization of Tōbyōki Blog Contents

**Figure 3:**
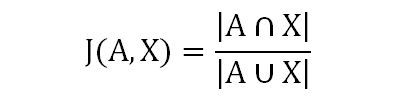
Calculation of the Jaccard coefficient.

Because visualization approaches could be useful for PV, we used all sentences containing at least one of the above 100 drugs to calculate Jaccard coefficients to measure the similarity between term pairs. Jaccard coefficients index the degree of cooccurrence between term pairs by showing how much the terms overlap. For instance, Figure 3 shows the calculation of the Jaccard coefficient for drug A and verb X [34].

Using these Jaccard coefficients, we visually represented the words associated with depression or RA in word clouds. In the word clouds, the size of each word reflected the frequency with which the word appeared in text (ie, the more frequently a word appeared, the larger the word was shown in the word cloud). The colors of each word were randomly assigned and did not have any meaning. Word clouds could be used in PV to achieve an initial, intuitive understanding of data. We also created a word cooccurrence network for patients with RA to evaluate the occurrence of words in conjunction with the names of 4 biological drugs approved for RA. Word cooccurrence network analysis could be used in PV to explore terms related to specific drugs.

Statistical software R, JMP software version 11.2.1 (SAS institute), and Microsoft Excel were used for the analysis.

### Ethics Approval

The study protocol was reviewed and approved by the nonprofit MINS Institutional Review Board [35]. The board waived informed consent because the data source did not contain personal information. In addition, we presented the data at the group level rather than at the individual level.

## Results

### Analysis A: Using the Whole TOBYO Database

#### Demographic Characteristics of the TOBYO Database

As of June 4, 2016, the *tōbyōki* blogs aggregated in the TOBYO database comprised 54,010 blogs representing 1405 disorders. The blogs were started from 1994 to 2016, but more than 90% of them were started from 2005 to 2015.

As shown in Table 2, information on gender could be identified in most of the blogs (99.60%, 53,794/54,010). More blogs were written by female bloggers (68.80%, 37,161/54,010) than by male bloggers (30.80%, 16,633/54,010). Of approximately 40% of *tōbyōki* blogs in the TOBYO database with information on age at onset, more than half were written by people less than 50 years old. The peak age at onset was 20-34 years (24.44%, 13,201/54,010), followed by 35-49 years (16.35%, 8830/54,010) and less than 20 years (16.16%, 8730/54,010).

**Table 2:** Distribution of gender and age at onset in TOBYO database.

Variable	Category	All, n (%)^a^	Male, n (%)^a^	Female, n (%)^a^	Unknown, n (%)^a^
**Gender**		54,010 (100)	16,633 (31)	37,161 (69)	216 (0)
**Age at onset of primary disorder^b^**	≤19 years old	8730 (16)	4045 (24)	4654 (13)	31 (14)
	20-34 years old	13,201 (24)	2726 (16)	10,460 (28)	15 (7)
	35-49 years old	8830 (16)	2450 (15)	6371 (17)	9 (4)
	50-64 years old	2048 (4)	1083 (7)	961 (3)	4 (2)
	≥65 years old	808 (2)	381 (2) 426 (1)	1 (1)	
	Unknown	20,393 (38)	5948 (36)	14,289 (39)	156 (72)

^a^Number of blogs equals number of patients. Because of rounding, total values for proportion are not always 100%.

^b^Age at onset of primary disorder was obtained by checking profile or introductory page of each blog.

**Figure 4:**
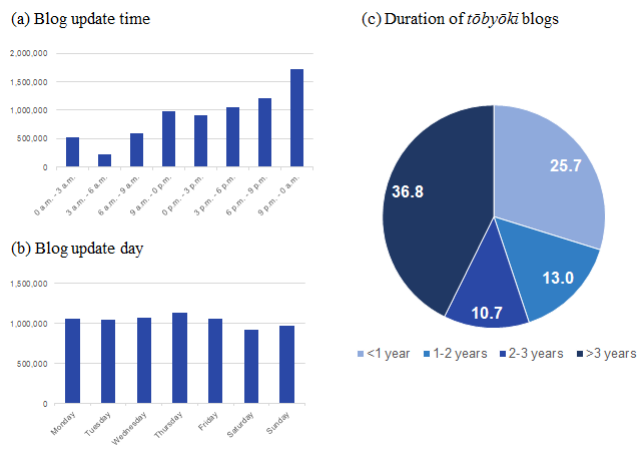
Comparison of population distribution between the TOBYO database and external databases.

We found apparent differences in population distribution between the TOBYO database and existing data sources such as the Japanese health insurance claims database, JADER database, and national population statistics (Figure 4). Compared with national statistics as a standard, the population in the TOBYO database tended to be younger and contained relatively more females than males. In contrast, the population of the JADER database was older with no particular gender differences between ages. The health insurance claims database did not include people older than 75 years, but data for the young to middle-aged group seemed to be abundant with no particular gender differences between age groups.

#### Distribution of Disorders in the TOBYO Database

As shown in Table 3, the most frequently appearing disorders in the TOBYO database were breast cancer (9.23%, 4983/54,010), depression (6.58%, 3556/54,010), infertility (4.50%, 2430/54,010), RA (2.07%, 118/54,010), and panic disorder (2.02%, 1090/54,010). These disorders were observed more frequently in females than in males in the TOBYO database. Categorization of disorders according to disease organ groups by a proprietary TOBYO classification system similar to MedDRA classification showed that the frequently appearing categories were neoplasms benign, malignant, and unspecified (31.20%, 16,851/54,010), psychiatric and behavior disorders (22.84%, 12,334/54,010), kidney, urological, or genital disorders (8.34%, 4507/54,010), and muscular, bone, or articular disorders (8.28%, 4471/54,010; Table 4).

**Table 3:** Primary disorders frequently described in the TOBYO database.

Name of disorders	Corresponding preferred term of MedDRA (version 19.1)	Gender: all, number of blogs and proportion among all disorders, n (%)^a^	Gender: male, number of blogs and proportion among each disorder, n (%)^a^	Gender: female, number of blogs and proportion among each disorder, n (%)^a^	Gender: unknown, number of blogs and proportion among each disorder, n (%)^a^
Breast cancer	Breast cancer	4983 (9)	6 (0)	4974 (100)	3 (0)
Depression	Depression	3556 (7)	1467 (41)	2077 (58)	12 (0)
Infertility	Infertility	2430 (5)	16 (1)	2411 (99)	3 (0)
Rheumatoid arthritis	Rheumatoid arthritis	1118 (2)	71 (6)	1045 (94)	2 (0)
Panic disorder	Panic disorder	1090 (2)	202 (19)	881 (81)	7 (1)
Schizophrenia	Schizophrenia	1024 (2)	336 (33)	683 (67)	5 (1)
Cervical cancer	Cervix carcinoma	934 (2)	0 (0)	933 (100)	1 (0)
Hysteromyoma	Uterine leiomyoma	802 (2)	0 (0)	802 (100)	0 (0)
Type 1 diabetes mellitus	Type 1 diabetes mellitus	792 (2)	188 (24)	602 (76)	2 (0)
Ulcerative colitis	Colitis ulcerative	683 (1)	253 (37)	428 (63)	2 (0)
Systemic lupus erythematosus	Systemic lupus erythematosus	665 (1)	33 (5)	631 (95)	1 (0)
Eating disorder	Eating disorder	664 (1)	11 (2)	651 (98)	2 (0)
Others		35,269 (65)			

^a^Number of blogs equals number of patients. Because of rounding, total values for proportion are not always 100%.

**Table 4:** Category for primary disorders frequently described in the TOBYO database.

Name of grouped category of disorders	Corresponding System Organ Class of MedDRA (version 19.1)	Gender: all, number of blogs and proportion among all disorders, n (%)^a^	Gender: male, number of blogs and proportion among each disorder, n (%)^a^	Gender: female, number of blogs and proportion among each disorder, n (%)^a^	Gender: unknown, number of blogs and proportion among each disorder, n (%)^a^
Neoplasms benign, malignant, and unspecified	Neoplasms benign, malignant, and unspecified (including cysts and polyps)	16,851 (31)	4196 (25)	12,621 (75)	34 (0)
Psychiatric and behavior disorders	Psychiatric disorders	12,334 (23)	4281 (35)	7998 (65)	55 (0)
Kidney, urological, or genital disorders	Renal and urinary disorders	4507 (8)	774 (17)	3710 (82)	23 (1)
Muscular, bone, or articular disorders	Musculoskeletal and connective tissue disorders	4471 (8)	648 (15)	3813 (85)	10 (0)
Congenital disorders or abnormal chromosome	Congenital, familial, and genetic disorders	2972 (6)	1455 (49)	1496 (50)	21 (1)
Neurological disorders	Nervous system disorders	2877 (5)	1171 (41)	1691 (59)	15 (1)
Endocrine, nutritional, or metabolic disorders	Metabolism and nutrition disorders^b^	2269 (4)	636 (28)	1626 (72)	7 (0)
Digestive system disorders	Gastrointestinal disorders	1876 (4)	773 (41)	1093 (58)	10 (1)
Circulatory conditions	Cardiac disorders	1523 (3)	933 (61)	580 (38)	10 (1)
Infections and infestations	Infections and infestations	849 (2)	480 (57)	357 (42)	12 (1)
Blood, hematopoietic, or immunological disorders	Blood and lymphatic system disorders	777 (1)	314 (40)	458 (59)	5 (1)	
Skin disorders	Skin and subcutaneous tissue disorders	755 (1)	230 (31)	521 (69)	4 (1)
Others		1949 (4)			

^a^Number of blogs equals number of patients. Because of rounding, total values for proportion are not always 100%.

^b^There was an another possible MedDRA coding: endocrine disorders.

#### Additional Characteristics of the TOBYO Database

**Figure 5:**
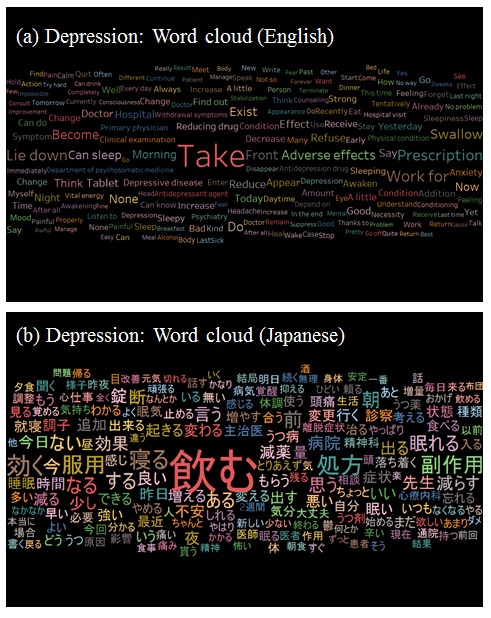
Additional characteristics of the TOBYO database.

We also highlighted unique data elements by analyzing behavioral characteristics of writing *tōbyōki* blogs and found that most writers updated their blogs between 9 PM and 0 AM (Figure 5). No particular patterns were observed according to which days of the week blog entries were posted. About 40% of the blogs in the TOBYO database (36.81%, 19,879) had continued for more than 3 years.

### Analysis B: Using Subset of Selected Disorders in the TOBYO Database

#### Mining Events Appearing in Tōbyōki Blogs

Comparison of depression (Table 5) and RA (Table 6) events in *tōbyōki* blogs and the JADER database showed apparent differences in the types and frequencies of events observed. Subjective, symptomatic terms and general terms for patients tended to be frequently observed in *tōbyōki* blogs (eg, anxiety, headache, and pain), whereas more technical, medical terms (eg, syndrome and abnormal laboratory test result) tended to be observed frequently in the JADER database. Exceptionally, the fact that “interstitial lung disease” in patients with RA was observed frequently in both *tōbyōki* blogs and the JADER database suggested relatively high attention for this event.

**Table 5:** Comparison of events in patients with depression in the TOBYO database and JADER database.

Medical terms observed in the TOBYO database				Medical terms observed in the JADER database		
Rank	Event description in the TOBYO database	MedDRA Preferred Terms in the TOBYO database	Number of events	Rank MedDRA Preferred Terms in the JADER database	Number of events	
1	Adverse reaction^c^	Adverse reaction	9977	1	Inappropriate antidiuretic hormone secretion	247
2	Depression	Depression	6218	2	Serotonin syndrome	226
3^a^	Anxiety	Anxiety	5695	3	Suicide attempt	213
4	Headache	Headache	2977	4	Seizure	153
5	Pain	Pain	2016	5	Completed suicide	152
6	Withdrawal symptom	Withdrawal syndrome	1848	6	Hyponatraemia	143
7	Stress	Stress	1194	7^a^	Mania	121
8	Malaise	Malaise	1162	8	Neuroleptic malignant syndrome	108
9^a^	Feeling queasy	Nausea	1140	9^a^	Dizziness	103
10	Constipation	Constipation	897	10^a^	Nausea	101
11	Psychosis^c^	Mental disorder	777	11	Aggression	100
12^a^	Dizzy	Dizziness	762	12	Suicidal ideation	96
13	Paroxysmal attack^c^	Seizure-like phenomena	691	13	Hepatic function abnormal	93
14	Emotional instability^c^	Affect lability	685	14^a^	Tremor	92
15	Abnormality	NA^b^	641	15	Altered state of consciousness	90
16	Suffering^c^	Sense of oppression	637	16	Activation syndrome^d^	86
17	Feeling down^c^	Depressed mood	619	17	Irritability	85
18	Sleep disorder	Sleep disorder	601	18	Loss of consciousness	78
19	Schizophrenia	Schizophrenia	479	19^a^	Anxiety	75
20	Allergy	Hypersensitivity	474	20	Hallucination	74
21^a^	Mania	Mania	472	21	Somnolence	69
22	Migraine	Migraine	445	22	Delirium	68
23	Pollinosis	Seasonal allergy	427	23	Liver disorder	61
24	Hypersomnia	Hypersomnia	418	24	Urinary retention	60
25	Bipolar disorder	Bipolar disorder	376	25	Overdose	58
26	Diarrhea	Diarrhea	368	26^a^	Intentional self-injury	57
27^a^	Trembling	Tremor	326	27	Electrocardiogram QT prolonged	56
28	Panic disorder	Panic disorder	314	28	Drug withdrawal syndrome neonatal	53
29	Asthma	Asthma	294	29	Insomnia	50
30	Weight increased	Weight increased	284		Intentional overdose	50
31	Diabetes mellitus	Diabetes mellitus	281			
32	Ache stomach	Abdominal pain upper	277			
33	Psychiatric disorder^c^	Mental disorder	266			
34	Vomiting	Vomiting	240			
35	Abdominal pain	Abdominal pain	237			
36	Slight fever	Pyrexia	231			
37	Influenza	Influenza	228			
38	Physical deconditioning	Asthenia	226				
39	Myalgia	Myalgia	225			
40	Dependence	Dependence	210			
41	Inflammation	Inflammation	206			
42	Panic attack	Panic attack	197			
43^a^	Self-injury	Intentional self-injury	194			
44	Hypertension	Hypertension	179				
45	Severe pain	Pain	152			

^a^Events appearing in both databases (the TOBYO database and JADER database).

^b^NA (not applicable) in columns for MedDRA PTs (preferred terms) means there was no corresponding term in MedDRA.

^c^Coding discrepancies occurred between the 2 reviewers (the different suggestions from the reviewers are shown in parentheses): Adverse reaction (adverse reaction or adverse drug reaction), Psychosis (mental disorder or psychotic disorder), Paroxysmal attack (seizure-like phenomena or seizure), Emotional instability (affect lability or feeling abnormal), Suffering (sense of oppression or emotional distress), Feeling down (depressed mood or emotional distress), and Psychiatric disorder (mental disorder or psychotic disorder).

^d^Activation syndrome is a generic term used for central nervous system stimulation symptoms that are potential adverse effects caused by selective serotonin reuptake inhibitors.

**Table 6:** Comparison of events in patients with rheumatoid arthritis in the TOBYO database and JADER database.

Medical terms observed in the TOBYO database				Medical terms observed in the JADER database		
#	Event description in the TOBYO database	MedDRA PTs in the TOBYO database	Number of events	#	MedDRA PTs in the JADER database	Number of events
1	Rheumatoid arthritis	Rheumatoid arthritis	9193	1^a^	Pneumonia	1109	
2	Pain	Pain	6471	2^a^	Interstitial lung disease	835
3	Adverse reaction^c^	Adverse reaction	4378	3	*Pneumocystis jirovecii* pneumonia	575
4	Inflammation	Inflammation	1044	4	Cellulitis	330
5	Swelling	Swelling	761	5	Sepsis	292
6	Feeling queasy	Nausea	661	6	Herpes zoster	255
7	Abnormality	NA^b^	440	7	Pyrexia	252
8	Headache	Headache	419	8	Pneumonia bacterial	216
9	Allergy	Hypersensitivity	385	9	Infusion-related reaction	215
10	Stress	Stress	380	10	Pulmonary tuberculosis	189
11	Collagen disorder	Collagen disorder	373	11	White blood cell count decreased	159
12	Diarrhoea	Diarrhoea	370	12	Arthritis bacterial	149
13	Unwell	Malaise	357	13	Lymphoma	147
14	Pollinosis	Seasonal allergy	340	14	Atypical mycobacterial infection	130
15	Severe pain	Pain	306	15^a^	Hepatic function abnormal	121
16	Osteoporosis	Osteoporosis	276	16	Pancytopenia	116
17	Muscle stiffness^c^	Musculoskeletal stiffness	272	17	Organizing pneumonia	104
18	Influenza	Influenza	261		Histiocytosis hematophagic	104
19	Infections	Infection	241	19	Pleurisy	99
20^a^	Tuberculosis	Tuberculosis	236	20	Platelet count decreased	98
21	Stomatitis	Stomatitis	218	21	Urinary tract infection	97
^a^	Pneumonia	Pneumonia	218		Disseminated tuberculosis	97
23	Asthma	Asthma	181	23	Disseminated intravascular coagulation	94
24	Eczema	Eczema	165	24	Breast cancer	88
25	Arthralgia	Arthralgia	162	25	Peritonitis	87
26	Slight fever	Pyrexia	147	26^a^	Tuberculosis	86
27	Dizzy	Dizziness	145		Pyelonephritis	86	
28^a^	Interstitial pneumonia	Interstitial lung disease	137	28	Septic shock	85
29	Numbness	Hypoesthesia	132	29	Neutrophil count decreased	79
30	Hemorrhage	Hemorrhage	116		Gastric cancer	79
31	Depressed mood^c^	Depressed mood	115			
32	Constipation	Constipation	115			
33	Suffering^c^	Sense of oppression	106			
34	Abdominal pain	Abdominal pain	103			
35	Diabetes mellitus	Diabetes mellitus	100			
36	Rash	Rash	99			
37	Moon face^c^	Cushingoid	98			
38	Edema	Edema	96			
39	Death	Death	96				
40	Itch	Pruritus	95			
41^a^	Hepatic function abnormal	Hepatic function abnormal	93			
42	Urticaria	Urticaria	92			
43	Vomiting	Vomiting	91			
44	Physical deconditioning	Asthenia	90			
45	Hyperthermia	Hyperthermia	89			
46	Paroxysmal attack^c^	Seizure-like phenomena	85			
47	Cataract	Cataract	84			

^a^Events appearing in both databases (the TOBYO database and JADER database).

^b^NA (not applicable) in columns for MedDRA PTs (preferred terms) means there was no corresponding term in MedDRA.

^c^Coding discrepancies occurred between the 2 reviewers (the different suggestions from the reviewers are shown in parentheses): Adverse reaction (Adverse reaction or Adverse drug reaction), Muscle stiffness (Musculoskeletal stiffness or Muscle rigidity), Depressed mood (Depressed mood or Listless), Suffering (Sense of oppression or Emotional distress), Moon face (Cushingoid or Face edema), and Paroxysmal attack (Seizure-like phenomena or Seizure).

#### Visualization of Contents in Tōbyōki Blogs

**Figure 6:**
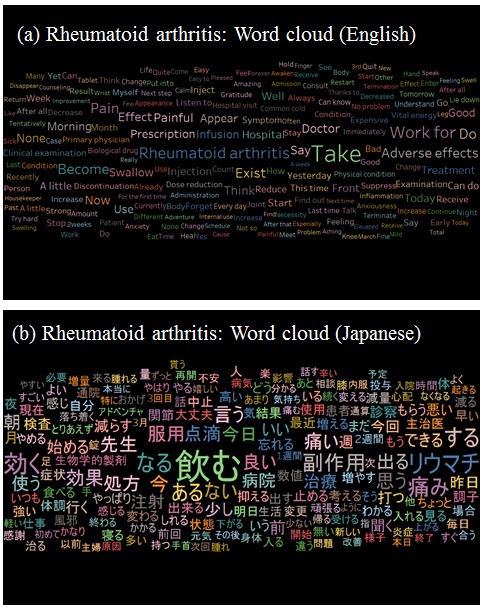
Word cloud: visualization of words frequently observed in tōbyōki blogs of patients with depression.(a) English version translated from the original Japanese and (b) the original Japanese version.

**Figure 7:**
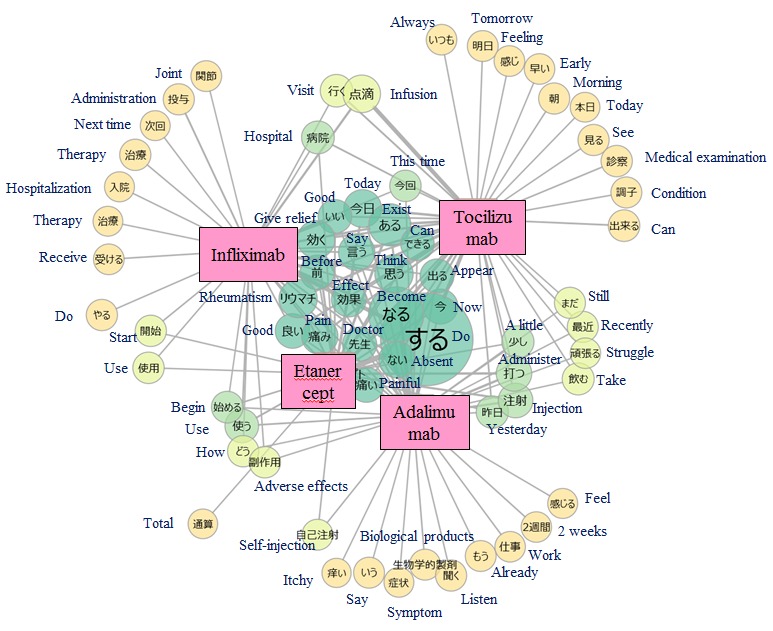
Word cloud: visualization of words frequently observed in tōbyōki blogs of patients with rheumatoid arthritis.(a) English version translated from the original Japanese and (b) the original Japanese version.

As depicted in Figures 6 and 7, “take” (as in “take medicine”) was the most frequent word in the datasets for depression and RA, suggesting that extraction of *tōbyōki* blog content containing the 100 most frequently mentioned drugs helped focus the data. Among patients with depression (Figure 6), sleep-related terms such as “lie down,” “sleep (noun),” “sleep (verb),” “sleepiness,” “awakening,” and “awaken” were observed, indicating that patients shared information about their disease conditions. We also found therapy-specific words such as “adverse effects,” “antidepressant agent,” “depression drug,” and “withdrawal symptoms.” Among patients with RA (Figure 7), pain-related terms such as “pain,” “painful,” “swelling,” and “stiffness” were frequently noted, indicating that these were important words for characterizing RA.

**Figure 8:**
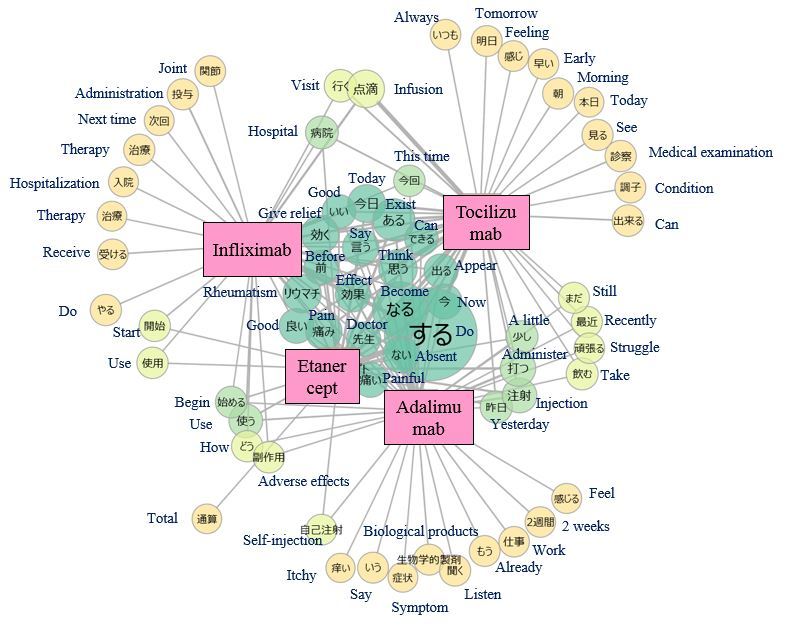
Word co-occurrence network: visualization of words occurring with biological drugs in tōbyōki blogs of patients with rheumatoid arthritis.Because the original language is Japanese, English translations are shown together.

As depicted in Figure 8, the words “rheumatism,” “give relief,” “pain,” and “painful” were located at the center of the word cooccurrence networks of the 4 biological drugs considered in this study, meaning that these words were frequently used in association with all 4 drugs. The characteristics of each drug were also observed in the margins of the word cooccurrence networks. For example, adalimumab and etanercept, administered as subcutaneous injections, were associated with the word “self-injection,” and infliximab and tocilizumab, administered as intravenous infusions, were associated with the word “infusion.”

## Discussion

### Principal Findings

Patient-generated data is likely to play a key role in improving PV [36]. In Japan, however, a system of self-reporting by patients is still being considered [10] and no patient-generated data resources have been explicitly identified. As one option for such a resource, this study evaluated the TOBYO database from the PV perspective.

In the whole TOBYO database, more blogs were written by female bloggers, and fewer blogs were written by people older than 50 years (Table 2). These findings were consistent with the results of a general survey of Internet usage in Japan [23]. Reflecting the fact that a higher percentage of *tōbyōki* blogs were written by women, the most frequently appearing disorders in the TOBYO database tended to have a higher prevalence in women: breast cancer, cervical cancer [37], RA [38], and panic disorder [39] (Table 3). Additional analysis of *tōbyōki* blogs would be more realistic for these disorders with a high prevalence in women. Our findings also suggested the relevance to frequently appearing disorders such as psychiatric disorders with subjective symptoms that have important clinical meaning, refractory disorders, autoimmune disorders, and other chronic disorders.

As shown in Tables 5 and 6, *tōbyōki* blogs written by patients with depression or RA contained symptomatic, subjective terms rather than the medical diagnosis or other medical terms. This revealed a difference between *tōbyōki* blogs and the JADER database generated by HCPs and implied that the TOBYO database might have the advantage of enabling the analysis of patient-level outcomes that could not be captured in existing data sources. Indeed, previous research has shown that psychiatric events are difficult to identify in health care administrative databases because physicians have difficulty detecting them and patients avoid reporting the symptoms to their physicians [40]. Another interesting possibility is that even if a patient reporting system is implemented, patients may not voluntarily report events that they do not consider to be AEs, as suggested by a previous research conducted on patients with Parkinson’s disease [41]. In such a situation, in which patients themselves do not consider the possibility of AEs, the TOBYO database can be useful for capturing initial symptoms as AE signals.

We confirmed the feasibility of analyzing patient narratives using text mining to draw insights from *tōbyōki* blogs. Word clouds suggested characteristic words associated with selected conditions, such as “sleeping” and “anxiety” with depression and “pain” and “painful” with RA. This suggested that *tōbyōki* blogs were a useful resource for understanding characteristic information for each disorder. We were also able to identify words commonly associated with the 4 biological drugs located at the center of the word cooccurrence networks (Figure 8). The common words revealed in this study were not particularly noteworthy, but further research using the same approach with different drugs or disease areas might be useful for exploring drug safety concerns such as unknown AEs. For example, a report analyzing tweets written by Japanese patients with cancer suggested that visualizing narratives with word cooccurrence networks could be a useful approach to obtain insights from social media [42].

We noted several strengths of *tōbyōki* blogs as a resource for data analysis in this study. One was the ease of obtaining patient background information as summarized in Table 2. In contrast to other data sources such as Web-based discussion forums in which patient background information was inherently limited [43], *tōbyōki* blogs usually had a profile or introduction page from which a substantial level of information could be collected. Another strength was that most *tōbyōki* bloggers wrote their blogs voluntarily to record and share their experiences with others, resulting in primarily subjective descriptions of patient experiences. This first-hand, observational quality, free from obligations or interventions, might enable researchers to better understand patients’ actual concerns. A third strength was that compared with common blogs or social media (even those written by patients), *tōbyōki* blogs might be more likely to contain analyzable information on health-related or life-related topics because serious disease and other health crises were typical motivations for starting *tōbyōki* blogs.

### Limitations

This study had several limitations. First, because *tōbyōki* blogs were written by only a segment of the patient population, generalization of the findings required caution. For instance, the elderly population might be underrepresented in Internet sources [23]. In addition, as a patient’s condition became more severe, it might be more difficult for them to continue writing their *tōbyōki* blogs. These biases should be considered when interpreting the results. Second, the insights obtained from qualitative text-mining approaches were based on some degree of subjective interpretation by researchers. For example, in word clouds, the relative size of each word reflected its frequency. It would be helpful to identify frequent or important words that were mentioned by many bloggers. On the other hand, because the size of each word did not reflect its clinical significance, it was possible that some smaller words might have greater clinical significances. Although word clouds have the potential to provide some insights from textual data, interpretation should be done in caution, keeping their pros and cons in mind. Third, some technical improvements would be necessary to extract more meaningful knowledge from the texts used in this study. For instance, we only considered fragmented words for analysis. By excluding phrases and other word combinations, we might have missed some important concepts or patient feelings. Additional techniques such as entity linking or named entity recognition should be considered in future studies to improve the results. Finally, because the language in social media tends to be highly informal and contain a wide variety of expressions, identification of specific concepts such as AEs and medicinal drugs from the unstructured narratives is a challenge. Although we could identify frequently appearing medical events in the TOBYO database, as shown in Tables 5 and 6, it is apparent that not all these events were AEs because we did not consider whether they had occurred before or after drug administration. Additional work is necessary to identify AEs occurring after drug administration.

### Future Challenges for Social Pharmacovigilance

We also recognized future challenges for the effective use of social media data in PV. First, there is a need for an official guidance or policy about the necessity of obtaining informed consent from patients and protecting privacy. Although research interest in the use of social media is growing, there is currently no consensus or guideline [44]. We think there is no need for artificial constraints such as obtaining subsequent informed consent for the use of blog data because they are already publicly available on the Internet. Regarding patients’ decisions on whether to share data, a study showed that patients in the cancer community tended to think positively about sharing as long as the benefit of sharing data outweighed the risk [45], and the authors recommended that researchers should be careful to protect patient anonymity. In accordance with this recommendation, we prepared all analysis output as summarized data and not individual-level data in consideration of patients’ rights to protected privacy. Second, we acknowledge that issues exist with the reliability and reproducibility of social media, particularly from the regulatory, good pharmacovigilance practice perspective. Concerns about the incorporation of false information have been noted previously [46]. Considering our study using *tōbyōki* blogs, we assume that the extent of this problem would not be very large because there is no conceivable incentive for maintaining a fake *tōbyōki* blog at this time. Selecting blogs with more than 10 pages in the screening process before registration in the TOBYO database would help to prevent the inclusion of fake blogs. Concerns about the reproducibility of analysis present a practical challenge. It is not realistic to keep a dynamic dataset that is updated every day and that may be updated retrospectively. To ensure the reproducibility of individual research, storing the final dataset as a snapshot is recommended. Finally, because the volume of data on the Internet is continuously growing, there may be a need to think about how to efficiently detect and process AE information on the Internet. One option is to improve the text-mining algorithm using dictionary-based methods by preparing an annotated corpus to recognize AEs and drugs. However, this process would be time-consuming and costly. Another option is the application of a machine learning approach by preparing a classifier algorithm that does not necessarily require the preparation of annotated corpora, and there has been a report of the application of a deep-learning technique to detect potential AEs from social media texts [47]. In summary, we need to tackle several practical and technical issues to efficiently incorporate social media resources into PV.

### Conclusions

PV activities should have a strong focus on patients’ actual experiences, concerns, and outcomes, and this approach is expected to uncover hidden AE signals earlier and help us understand AEs in a patient-centered way. This study described the fundamental characteristics of *tōbyōki* blogs in the TOBYO database and provided insights into considering the use of such data for PV. Specific application possibilities for the TOBYO database include the analysis of disorders with a high prevalence in women, psychiatric disorders with important subjective symptoms, refractory disorders, and other chronic disorders. Further research would facilitate the enhancement of PV by incorporating patient-generated data from the Internet.

## Abbreviations

AE: adverse event
ADR: adverse drug reaction
HCP: health care professional
ICSR: individual case safety report
JADER: Japanese Adverse Drug Event Report
MedDRA: Medical Dictionary for Regulatory Activities
PV: Pharmacovigilance
RA: rheumatoid arthritis
